# On the effect of the inlet configuration for anaerobic digester mixing

**DOI:** 10.1007/s00449-021-02617-4

**Published:** 2021-07-21

**Authors:** Soroush Dabiri, Johannes Sappl, Prashant Kumar, Michael Meister, Wolfgang Rauch

**Affiliations:** 1 Unit of Environmental Engineering, University of Innsbruck, Innsbruck, Austria; 2 Department of Environmental, Process, and Energy Engineering, Management Center Innsbruck, Innsbruck, Austria

**Keywords:** Computational fluid dynamics, Multi-phase flow simulation, Dead volume, Anaerobic digestion

## Abstract

Sludge recirculation mixing in anaerobic digesters is essential for the stable operation of the digestion process. While often neglected, the configuration of the sludge inlet has a substantial influence on the efficiency of the mixing process. The fluid is either injected directly into the enclosed fluid domain or splashes onto the free surface of the slurry flow. In this paper, the aim was to investigate the effect of the inlet configuration by means of computational fluid dynamics—using ANSYS Fluent. Single-phase and multi-phase models are applied for a submerged and splashing inlet, respectively. To reduce the high computational demand, we also develop surrogate single-phase models for the splashing inlet. The digester mixing is analyzed by comparing velocity contours, velocity profiles, mixing time and dead volume. The non-Newtonian characteristics of the sludge is considered, and a $k-\varepsilon$ model is employed for obtaining turbulence closure. Our method is validated by means of a previous study on the same geometry. Applying a submerged inlet configuration, the resulting dead volume in the tank is estimated around 80 times lower than for the case of a splashing inlet. Additionally, by emulating the multi-phase model for splashing inlet configurations with a single-phase one, the simulation clock time reduced to 15%.

**Supplementary Information:**

The online version contains supplementary material available at 10.1007/s00449-021-02617-4.

## Introduction

Anaerobic digestion (AD) is a widely established biological process to both stabilize organic waste and generate biogas. Today biogas (approx. two thirds being methane) is widely used as a source of renewable energy [1], as well as for the production of organic acids and hydrogen [2].

The efficiency of the AD process in the tank depends on a variety of geometric features (like the dimensions and shape of the tank, the geometry of in- and outlet, mixing devices, etc.) and operating conditions (feeding pattern, temperature regime, operation of mixing devices, etc.). Especially the mixing conditions (and subsequently the flow field) are important, since only proper agitation of the slurry flow leads to a close contact between active microorganisms and feed substrates and thereby enhances the mass transfer within [3, 4].

Thus, detailed information on the temporal and spatial dynamics of the flow velocities inside the tank are essential for an optimized operation. However, it is due to the structural limitations of digesters that experimental measurements in the tank itself are de-facto impossible. As an alternative, computational fluid dynamics (CFD) [5] methods are applied to study digester mixing. It is common understanding that CFD offers a robust approach to investigate fluid flow properties, e.g. velocity fields, turbulence, particle trajectories, rates of energy dissipation. Likewise, CFD simulations help to determine zones with insufficient mixing intensity.

In the literature already a variety of investigations are found that exemplify methods for applying CFD modeling in both AD research and (real-world) operation. It is due to the high importance of the mixing process [6] that CFD models are also employed as a practical method to evaluate the efficiency of the mixing devices itself. An exhaustive review of CFD modeling in bioreactors is found in [7]. Due to its robustness, the Finite Volume Method (FVM) has established itself as the preferred numerical concept to solve the equations of fluid dynamics.

Mechanical mixing by means of suitable devices such as propellers, etc. is a key process in digester mixing. Various aspects of modelling stirred tanks were investigated in the past, e.g. turbulence enclosures, velocity gradient [8, 9], mixing rate, mixing type [10, 11] and shear stress [12, 13]. Zhang et al. [14] analyzed the flow field and power consumption in stirred digester tanks with different feedstocks, considering the specific non-Newtonian characteristics in each digester. In order to optimize the net energy consumption, they proposed a ratio of two different feedstocks for anaerobic digestion. Lebranchu et al. [13] used the software ANSYS Fluent to investigate the effect of shear stress on methane generation in a digester, which is stirred by a helical ribbon mixer. They validated their simulation with power numbers from rheology experiments, and they defined a criterion for the maximum range of shear stress in the digester. Meister et al. [11] investigated a full-scale egg shaped digester, where both sludge recirculation and an impeller within a draft tube contributed to the mixing. Analyzing the turbulent flow field as a function of total solids (TS) concentration, they proposed an increase in the range of impeller agitation speed for the cases with higher TS concentrations. Rezavand et al. [36] studied the same model as Meister et al., although they analyzed the recirculation and the draft-tube mixer within a fully Lagrangian computational platform. The two works in [11] and [36] are further elaborated in the Supplementary material file—Sect. 5. An important parameter to assess the quality of mixing is to estimate the inactive volumes, also known as dead zones or dead volumes, respectively. Such zones are defined as regions where the velocity magnitude is below a specified criterion [9, 15]. Low et al. [16] suggests to reduce the amount of the dead volume by improving the mixing on the basis of model predictions. Collivignarelli et al. [17], on the other hand, suggested localizing dead volume within AD tanks. For a case of mechanical AD mixing, Bridgeman [9] defined the dead volume—according to Vesvikar and Al-Dahhan [18]—as the regions where the velocity is less than 5% of the maximum velocity in the tank. Subsequently, they calculated the dead volume for different mixing speeds and for various TS concentrations. Another criterion to calculate the dead volume is defined by Hurtado et al. [15], denoting regions with a velocity magnitude below 0.02 m*/s* as dead volume. In their paper they concluded that improved mixing can decline the dead volume to 1–6%.

For continuously stirred tank reactors, the residence time distribution [19] and the turbulence intensity [18] were analyzed in addition to the dead volume. However, Dapelo and Bridgeman [20] defined a non-diffusive source field to evaluate the mixing quality by the use of uniformity index, which is more helpful to estimate mixing conditions as compared to the dead volume estimation. One reason is that their method also allows to calculate the mixing time, which is an important parameter to estimate the time needed for suitable recirculation of digesters. The mixing time is defined as the time needed for a homogeneous distribution of a tracer within the tank [21]. Mao et al. [21] used a converged model of an anaerobic digester, wherein they injected a tracer at the top of the digester. Observing tracer concentration after several minutes, they estimated the mixing time according to the method introduced by Wu [22].

Aguilar et al. [23] analyzed the effect of inlet mass flow rate on the mixing quality for the case of a pilot-scale egg-shaped digester which is only mixed by sludge recirculation. The mixing time is estimated based on the tracer concentration at the outlet cross-section—a method that has previously been implemented by Meroney and Colorado [24] and later by Hurtado et al. [15]. However, in this paper the residence time has been analyzed for up to 500 s only, which does not hold true for conditions in real-scale digesters.

Based on the literature, one can categorize the mixing approaches in AD tanks as mechanical mixing (by means of rotating devices), gas injection [15, 23, 25] and sludge recirculation. While the two former methods need complex devices/operation and cause high energy consumption, sludge recirculation is both simple and energy efficient [26]. Thus, it is a commonly used technique to supplement mechanical mixing or gas injection, but also applied as single mixing procedure.

An important aspect is the configuration of the inlet for sludge recirculation [23], which can be subdivided into a) splashing (free fall of the inflowing sludge) and b) submerged inlets. While the practical implications of inlet conditions are clear to both wastewater treatment plant operators and designers, a systematic evaluation of the differences and effects to the mixing conditions by means of CFD is hardly found in the literature. Leonzio [27] studied the nozzle configurations for injecting sludge within a cylindrical digester tank in real scale. However, in his study he focused on steady state hydrodynamics. Therefore, despite analyzing velocity contours, velocity gradient and power consumption, no tracer test was done in his study. López-Jiméneza et al. [28], investigated also the configurations of injectors, as well as dead volume and power consumption for sludge recirculation. Again, the simulations were conducted only in steady state and without any tracer tests. In this paper we go beyond present research and focus on the transient state of hydrodynamics, simulation of tracer experiments and the assessment of single-phase models for emulating two-phase models in simulation inlet configurations.

Summarizing, this paper aims to investigate the effect of two inlet configurations (splashing and submerged) on the digester hydrodynamics, by means of rigorous analysis of a suite of CFD simulations. This helps in understanding the capability of each inlet configuration for sludge recirculation, in terms of the energy consumption and mixing quality. Second, by identifying single-phase simulation models that are suitable to emulate two-phase model of splashing sludge, we aim to reduce the computational effort significantly. The outlet configuration is assumed as constant, since structural limitations does not allow changes in the outlet configuration. The submerged inlet is modeled by a single phase fluid (slurry flow), while the splashing inlet configuration is modeled in both multi-phase and single-phase. Additionally, the simulations are performed for both transient and steady state conditions. The commercial CFD code ANSYS Fluent v19 is used herein, which calculates the flow dynamics based on the Eulerian Finite Volume Method. The viscosity of the sludge is considered as a function of shear stress (i.e. applying non-Newtonian characteristics), based on suggestions in [20]. Also, a fluid-based tracking method is presented to estimate the mixing time.

## Materials and methods

### Geometry and Mesh

**Fig. 1:**
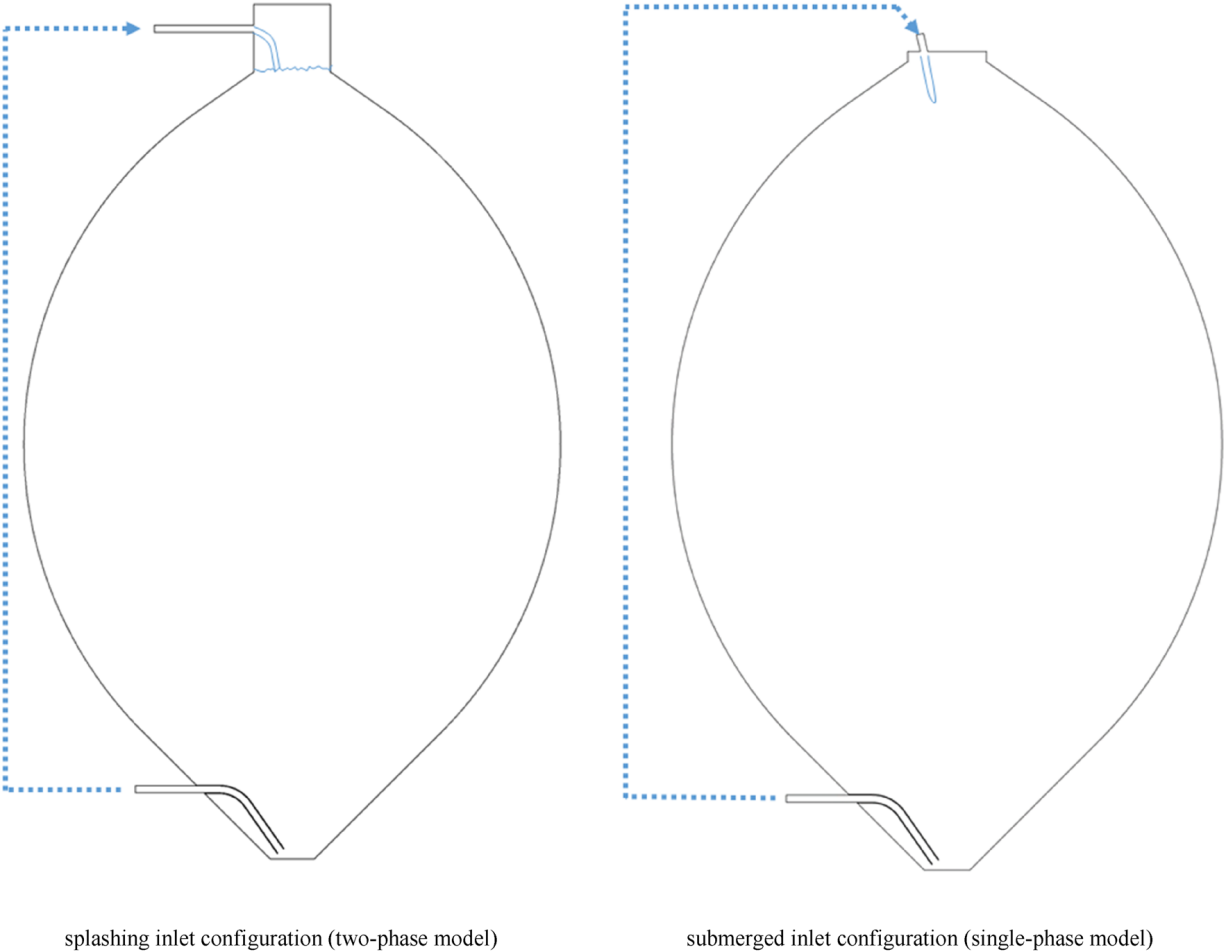
Inlet configurations of the anaerobic digester of the AIZ wastewater treatment plant

**Fig. 2:**
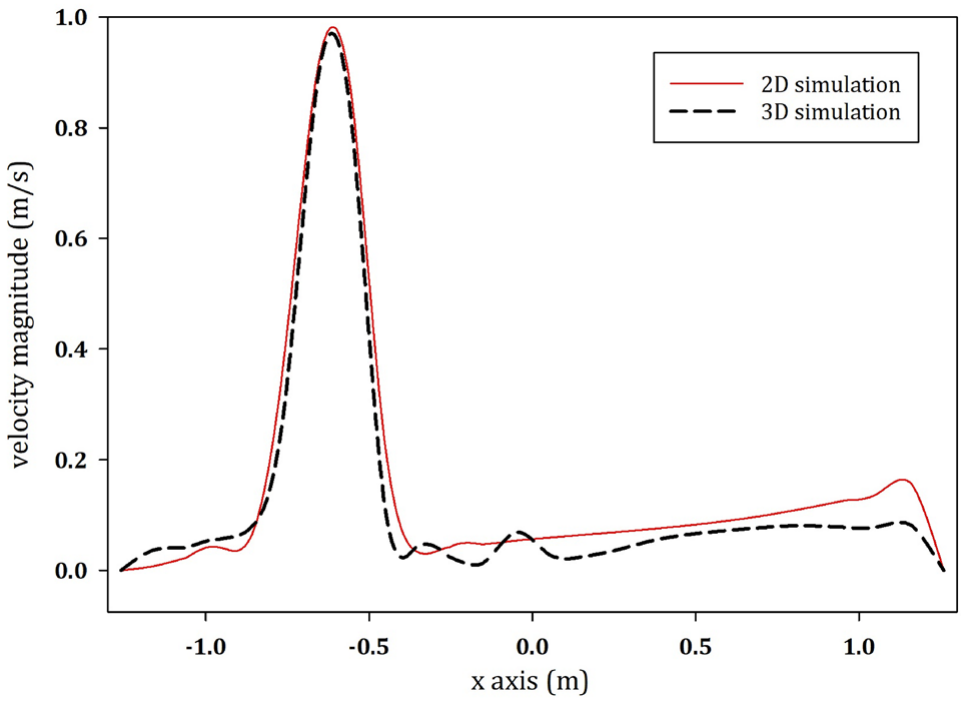
Vertical velocity magnitude along the horizontal axis of the 2D and 3D models 80 min after the initiation of the experiment

The investigations were based on a real case study i.e., a wastewater treatment plant (WWTP) in Tyrol, Austria. A sketch of the egg-shaped tank is shown in Fig. 1. A 2D simulation was conducted, since transient simulations in three dimensions of real digester with a two-phase model is computationally too expensive other than for unrealistic short time periods. We are aware that the results of the 2D and 3D simulations do not fully match together, however, a comparison between 2 and 3D single-phase models was conducted, for the upper part of the digester in the submerged inlet. The model was built on both 2D and 3D platforms. The velocity profiles at the cross-Sect. 30 cm below the sludge surface have been obtained for both models and plotted in Fig. 2 (for more information see the Supplementary material file—Sect. 4). These two profiles agree well and demonstrate the suitability of the approach in the upper part of the tank.

The maximum diameter of the tank is 15.4 m and the height of the sludge is 22.9 m. For both the splashing and submerged configurations, the inlet tube has a diameter of 0.2 m and is located at the top of the digester. The outlet tube, which also has a diameter of 0.2 m, is installed at the bottom of the digester. In the digester there are also three 0.1-m weir pipes close to the walls, the effects of which can be neglected, because of their small size in the real-scale (the locations of weir pipes are shown in the supplementary material file—Sect. 1).

The mesh for the FVM was implemented using the ANSYS Meshing tool, employing the mesh size option included for enhancing the quality of the triangular grid. The final mesh consisted of 404,878 elements for the single-phase models and 599,955 elements for the two-phase model. Grid characteristics are depicted in Table 1, and the actual grid network can be found in the Supplementary material file—Sect. 1).

**Table 1:** Mesh quality of the models

Model	Skewness	Aspect ration	Element quality
Multi-phase (splashing inlet)	Min: 7.43 e-10 Max: 0.66 Average: 0.05	Min: 1 Max: 2.57 Average: 1.07	Min: 0.6 Max: 1 Average: 0.98
Single-phase (splashing inlet)	Min: 1.27 e-04 Max: 0.52 Average: 0.03	Min: 1 Max: 1.95 Average: 1.02	Min: 0.49 Max: 0.99 Average: 0.97
Single-phase (submerged inlet)	Min: 5.04 e-05 Max: 0.78 Average: 0.05	Min: 1 Max: 2.96 Average: 1.08	Min: 0.43 Max: 0.99 Average: 0.96

### CFD Solver

In order to compute the transport of the feedstock material within the digester, the governing equations were solved in both transient and steady state for the slurry flow, which was treated as an incompressible fluid, utilizing ANSYS Fluent software, where the SIMPLE algorithm was employed for the discretization of the equations. Parallelization in the calculations was applied to improve the computational performance of the transient simulation. By doing so, the mesh and the data were split up into five partitions (based on our CPU capacity), and each mesh partition was allocated to a different compute process (or node). More information about this method can be found in [29].

### Governing equations

For all fluid flows the software solves the conservation equations for both mass and momentum, which are referred to as continuity (Eq. 1) and momentum (Eq. 2) equations, respectively [30].

1 $$\frac{\partial \rho }{{\partial t}} + \mathop{\nabla }\limits^{\rightharpoonup}  \cdot \left( {\rho \vec V} \right) = 0$$

2 $$\frac{{\partial \left( {\rho \vec V} \right)}}{\partial t} + \mathop{\nabla }\limits^{\rightharpoonup}  \left( {\rho \vec V\vec V} \right) = - \mathop{\nabla }\limits^{\rightharpoonup}  {\text{p}} + \mathop{\nabla }\limits^{\rightharpoonup}  \cdot \left( {\overline{\overline \tau } } \right) + \rho \vec g + \vec F,$$

where $\rho$ is the density and $\overrightarrow{V}$ is the velocity of the fluid. The first term in both equations represents the time-dependency of the solution for transient simulations; otherwise this term is equal to zero. The variable $\mathrm{p}$ denotes the static pressure, $\overrightarrow{F}$ is the additional force, which—in the two-phase case—is the momentum source term derived from surface tension and $\overline{\overline \tau }$ is the stress tensor. For turbulence closure, the RNG $k - \varepsilon$ model is selected herein because of its general ability to predict the flow field of turbulent flows. The option “scalable wall functions” is used to improve the accuracy of the k-ε model for calculating the equations in near-wall locations [31, 32].

In case the fluid involves a mixture of two different liquids for fluid tracking, the model also solves the transport equation. The solver estimates the local mass fraction of each liquid ${m}_{i}$ by solving a convection–diffusion equation for the $i$ th fluid. This conservation equation takes the following form as Eq. 3 [33]:

3 $$\frac{\partial }{\partial t}\left( {\rho {m_i}} \right) + \mathop{\nabla }\limits^{\rightharpoonup}  \cdot \left( {\rho \vec V{m_i}} \right) + \mathop{\nabla }\limits^{\rightharpoonup}  \cdot \vec J = 0,$$

where $\overrightarrow{J}$ is the mass diffusion term of the liquids (Since the tracer liquid has the same characteristics as the main liquid, the diffusion terms are equal. This will be further explained in Sect. 2.5). The way Eqs. 1 and  2 are written is based on the single-phase solver, while for the two-phase solver each quantity is estimated considering volume fractions, which increases the amount of calculations. Here, for modelling in two-phase condition, the volume of fluid (VOF) method is used, which is a surface-tracking technique applied to a fixed Eulerian mesh. In the VOF model, a single set of momentum equations is shared by both fluids, and the volume fraction of each fluid in each computational cell is tracked throughout the domain [29, 33]. As an example, the equation for continuity of the liquid phase including the liquid volume fraction in a two-phase set-up, is rewritten as Eq. 4:

4 $$\frac{1}{{{\rho_{liq}}}}\left[ {\frac{\partial }{\partial t}\left( {\alpha {\rho_{liq}}} \right) + \mathop{\nabla }\limits^{\rightharpoonup}  \cdot \left( {\alpha {\rho_{liq}}{{\vec V}_{liq}}} \right)} \right] = 0,$$

where $\alpha$ is the volume fraction of the liquid phase. The solver computes the governing equations in the two-phase model in an explicit scheme for accuracy and better interface resolution [34].

### Physical model

The density of the slurry flow was estimated as 1001.7 kg*/m*^*3*^ [35], and the non-Newtonian power law model was used to calculate the viscosity of the fluid. The non-Newtonian behavior of the feedstock was determined by the *TS* concentration. In our case, the fluid inside the digester of a municipal wastewater treatment plant had a *TS* concentration of about 5%. Thus, the non-Newtonian characteristics of the fluid at a mesophilic temperature of 35 *℃* are summarized in Table 2 [35, 36].

**Table 2:** Rheological properties of the slurry flow within the digester (original data from [35])

$TS \left(\mathrm{\%}\right)$	$K \left(Pa {s}^{n}\right)$	$n$	$\dot{\gamma }\left({s}^{-1}\right)$	${\eta }_{min} \left(Pa s\right)$	${\eta }_{max} \left(Pa s\right)$
5.4	0.192	0.562	50–702	0.01	0.03

The apparent viscosity of the rheological fluid depends on the shear rate. According to the power law equation, the viscosity is described as Eq. 5:

5 $$\eta =K{\dot{\left(\gamma \right)}}^{n-1},$$

where $\eta$ denotes the apparent viscosity,$\dot{\gamma }$ is the shear rate, $K$ represents the consistency index and $n$ indicates the flow behavior index which should be below one for pseudo-plastic fluids.

### Boundary and zone conditions

**Fig. 3:**
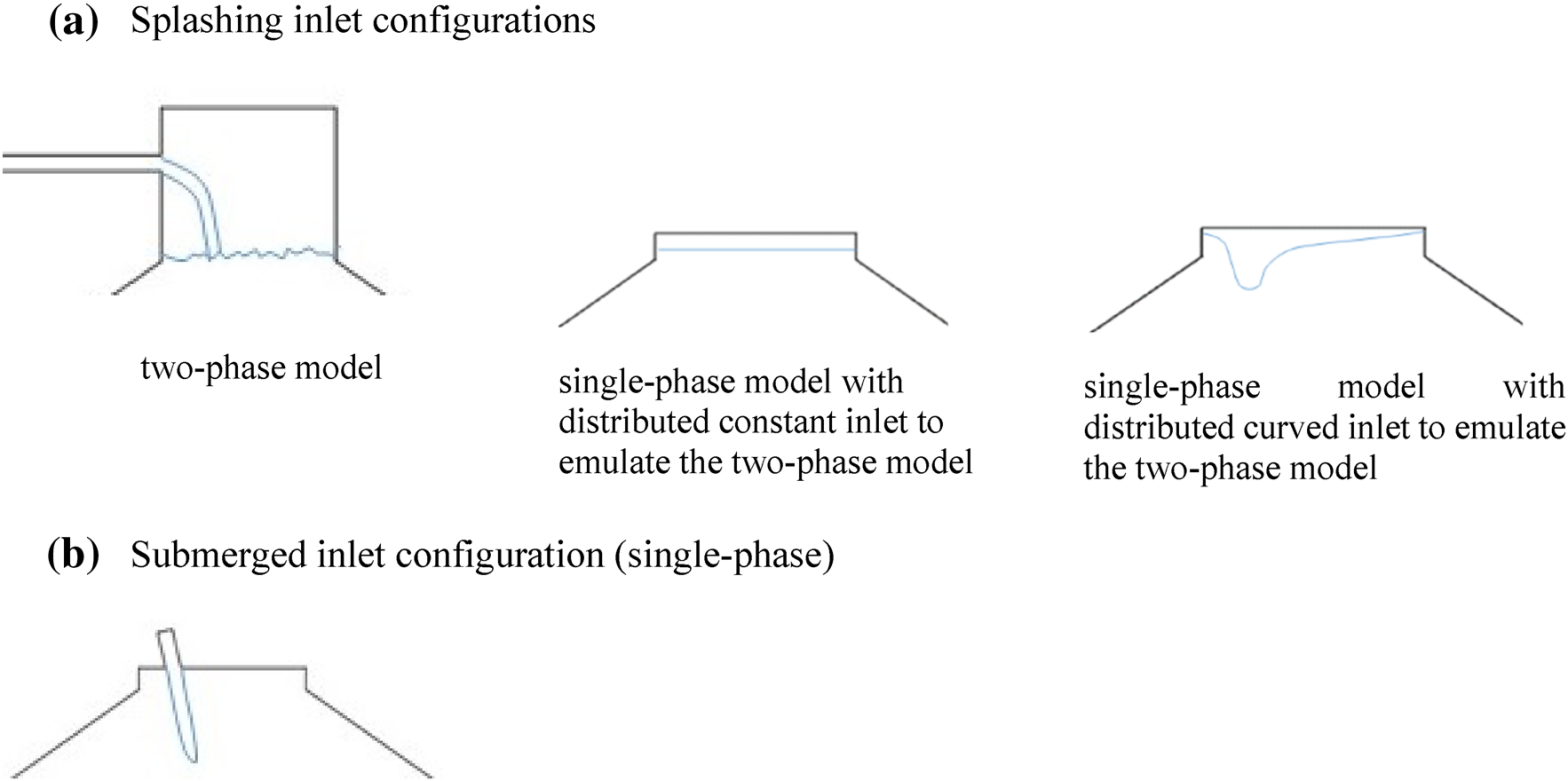
Sketches of the inlet configurations for **a** splashing inlet BCs and **b** submerged inlet BCs

Since the flow is supposed to enter the digester through either a splashing or a submerged inlet configuration, different inlet boundary conditions (BCs) were designed. For the splashing inlet configuration, a two-phase model (liquid–gas) was implemented, where the sludge falls freely on the liquid surface from the top. In order to emulate the above mentioned two-phase splashing inlet model for the sake of computational effort, two single-phase models were designed additionally. The first one mimics the splashing inlet with a distributed constant inlet configuration over the whole sludge surface, while the second one applies a curved inlet configuration. In both configurations the sludge enters with the same mass flow as in the submerged model (Fig. 3(a)). For simulating the submerged inlet configuration, only a single-phase model is necessary, as the sludge is directly injected into the calculation domain (Fig. 3b) without interaction with the gas phase.

Regarding the model with a distributed curved inlet configuration, the actual form of the curved inlet velocity profile was based on the results of the detailed two-phase simulation. The velocity profile below the sludge surface has been extracted and imposed to the single-phase model as a projected input velocity profile. An eight-degree polynomial represented properly the curve of the velocity inlet profile. A DEFINE_PROFILE macro function made it possible to access the velocity vectors at the centroid of a thread of inlet faces and looping over them (this procedure is declared in Supplementary material file—Sect. 2). At the walls of the digester, no-slip boundary conditions were imposed.

As for the initial conditions (IC), in the two-phase simulation, the calculation domain was divided into two zones. The lower one—from the bottom to the sludge surface—corresponds to the slurry flow and the upper zone to the gas phase (Supplementary material file—Sect. 1). Also, in order to track flow recirculation, in the single-phase models, a circular tracer region of another fluid—with the same density and viscosity-related characteristics as the main fluid (density of 1001.7 kg/m^3^ and the viscosity according to [36]), which constitutes 0.12% of the calculation domain volume—was initiated. Observing the distribution of this liquid (representing a tracer) in the tank enables the estimation of the initial mixing.

### Grid analysis

For mesh-based CFD models it is important to check the sensitivity of the results towards a change in grid spacing. In the present study, the grid convergence index (GCI) was computed by conducting a series of the grid refinement solutions. The probed parameter was the velocity magnitude at a central location of the tank (here at the maximum cross-section of the tank) in steady-state conditions. Based on [11, 37], three different mesh networks with different mesh sizes are tested (100,644; 404,878 and 1,117,855 elements for the distributed inlet configuration). The mesh size, *h*, is defined as in Eq. 6:

6 $$h = {\left( {\frac{1}{N}\mathop \sum \limits_{i = 1}^N {A_i}} \right)^{{\raise0.7ex\hbox{$1$} \!\mathord{\left/
 {\vphantom {1 2}}\right.\kern-\nulldelimiterspace}
\!\lower0.7ex\hbox{$2$}}}}$$

where $${A}_{i}$$ represent the area of the ith cell element, N represents the total number of cells within the calculation domain.$$h$$ is calculated for each grid, and the grid size ratio for every sequent grids should be greater than 1.3 ($$\left( {{r_{ij}} = {\raise0.7ex\hbox{${{h_j}}$} \!\mathord{\left/
 {\vphantom {{{h_j}} {{h_i}}}}\right.\kern-\nulldelimiterspace}
\!\lower0.7ex\hbox{${{h_i}}$}} > 1.3} \right)$$). The difference in velocity magnitudes of a specific point in different grids is calculated as $${\varepsilon }_{ij}={\varphi }_{j}-{\varphi }_{i}$$ , where $${\varphi }_{i}$$ and $${\varphi }_{j}$$ represent the solution parameter (here it is the velocity magnitude), probed for testing the ith and jth mesh grid, respectively. Then the apparent order of the method is calculated as in Eq. 7:

7 $$p=\frac{1}{\mathrm{ln}({r}_{ij})}\left|\mathrm{ln}\left|\frac{{\varepsilon }_{i+1,j+1}}{{\varepsilon }_{ij}}\right|+\mathrm{ln}(\frac{{r}_{ij}^{p}-1}{{r}_{i+1,j+1}^{p}-1})\right|$$

Afterwards, the relative error is calculated from Eq. 8:

8 $${e}_{a}^{ji}=\left|\frac{{\varphi }_{j}-{\varphi }_{i}}{{\varphi }_{i}}\right|$$

And finally the fine-grid convergence index is calculated as in Eq. 9:

9 $${GCI}_{fine}^{ji}=\frac{1.25{e}_{a}^{ji}}{{r}_{ji}^{p}-1}$$

According to the analysis (data are found in Supplementary material file—Sect. 3), for the case with distributed inlet configuration, the final ${GCI}_{fine}^{ji}$ is equal to 0.83%, which is the numerical uncertainty in the fine-grid solution for the selected parameter. Thus, the GCI value for the velocity magnitude at the specified point is very low. After evaluating GCI, the grid independence of the velocity magnitude profile should be verified. Thus, the velocity profile at the central cross-section of the digester (where the diameter is maximum) is analyzed for the three mesh networks (details are found in Supplementary material file—Sect. 3). The results reveal a difference of only 3.6% between the profiles of 404,878 and 1,117,855 elements. Therefore, it is concluded that the grid network with 404,878 elements has the proper mesh quality. A similar procedure is implemented for the model with the sub-merged inlet.

## Results

### Model validation

**Fig. 4:**
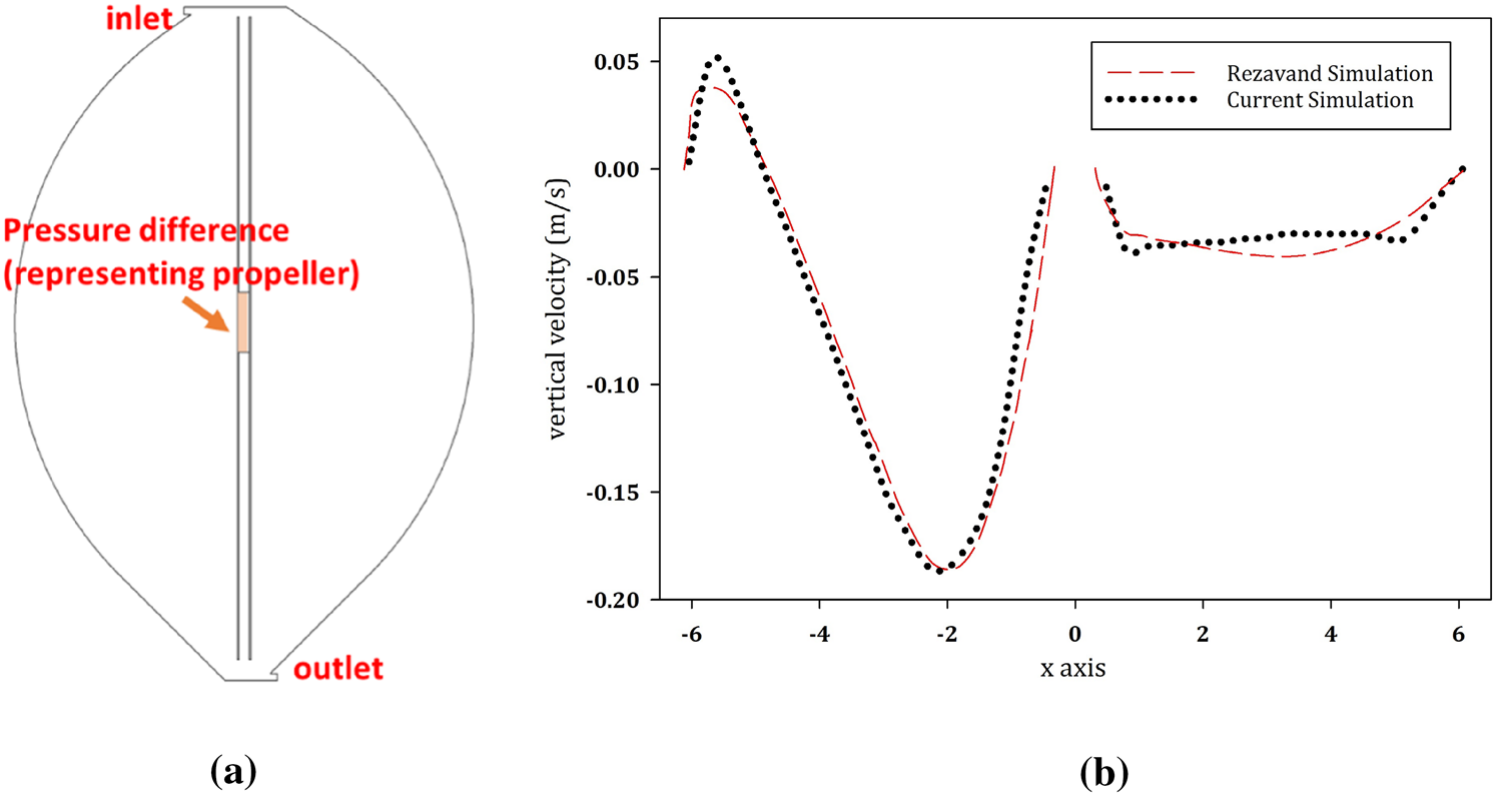
Left: the model including the draft tube for validation (**a**) and Right: the vertical velocity magnitude along the horizontal axis located at 6 m above the bottom of the reactor as the results of the validation (**b**)

For the validation of the method, the model derived herein is used to simulate a digester of similar geometry, but with different mixing conditions and inlet configurations. The model, shown by Fig. 4(a), applies a pressure difference representing the draft tube propeller device for mixing and has been previously analyzed in [36].

After setting similar initial and BCs, as well as applying a non-Newtonian fluid characteristics for 12.1% of *TS* (the chosen parameter value in [36]), in Fig. 4(b) the velocity profile 6 m above the bottom of the digester is evaluated and compared to the profile found in [36].

Figure 4(b) shows that the result of the model is in good agreement with Rezavand et al. [36]. The small mismatching parts are due to a different turbulence enclosure sub-model, as well as the different representation of the mixer in the two-dimensional models, which is done in our 2D simulation through an inserted pressure gradient.

**Fig. 5:**
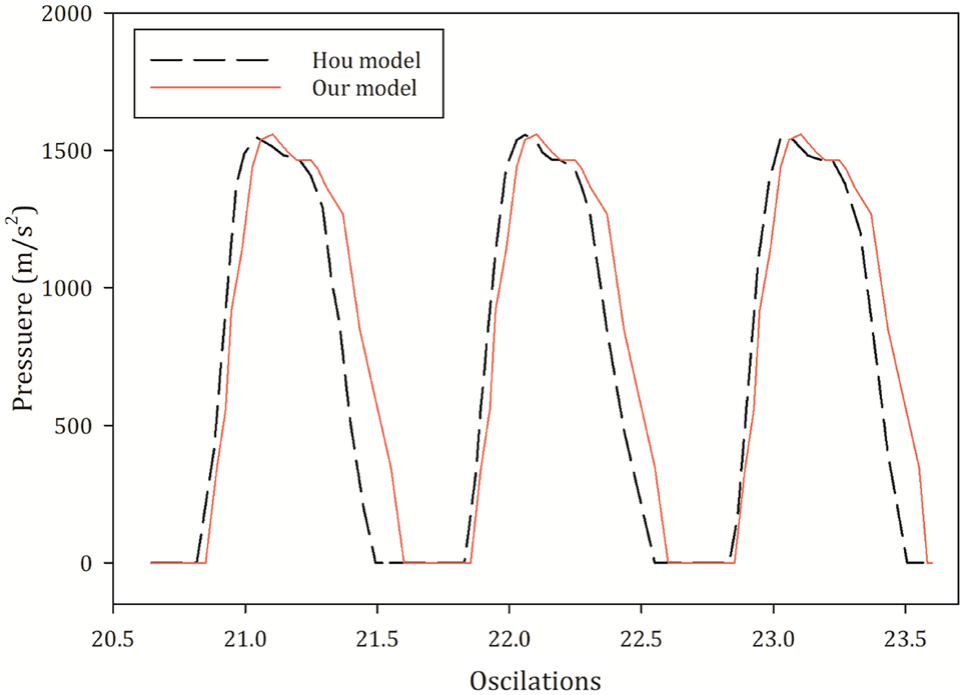
The pressure history at a specific point during 21st and 23rd oscillations within the two-phase calculation domain, based on our results and the results of Hou et. al

In order to validate the multiphase simulation, we have used the results of Hou et al. [38], as a similar software is used and likewise a multiphase method (VOF). The objective of their work was to adopt a two-phase model, in order to capture the sloshing motion of the free surface within a liquid tank. After developing our method based on their rectangular calculation domain, declared in [38], similar external oscillation with the same rate and displacement amplitude was applied to the tank. Then our results were compared to each other, as shown in Fig. 5.

Figure 5 depicts the pressure timeline at a specific point of the calculation domain between the 21st and 23rd oscillations for both our model and the model of Hou et al. Only a 4.3% of difference was observed, which is due to the use of RNG k-Epsilon turbulence closure in our model, while Hou et al. used the standard one.

### Multi-phase model

In order to investigate the splashing inlet characteristics, multi-phase simulation is run in transient. The results of this model are obtained as fluid phase volume fraction contours, as well as velocity profiles in the cross-section at the center of the tank.

**Fig. 6:**
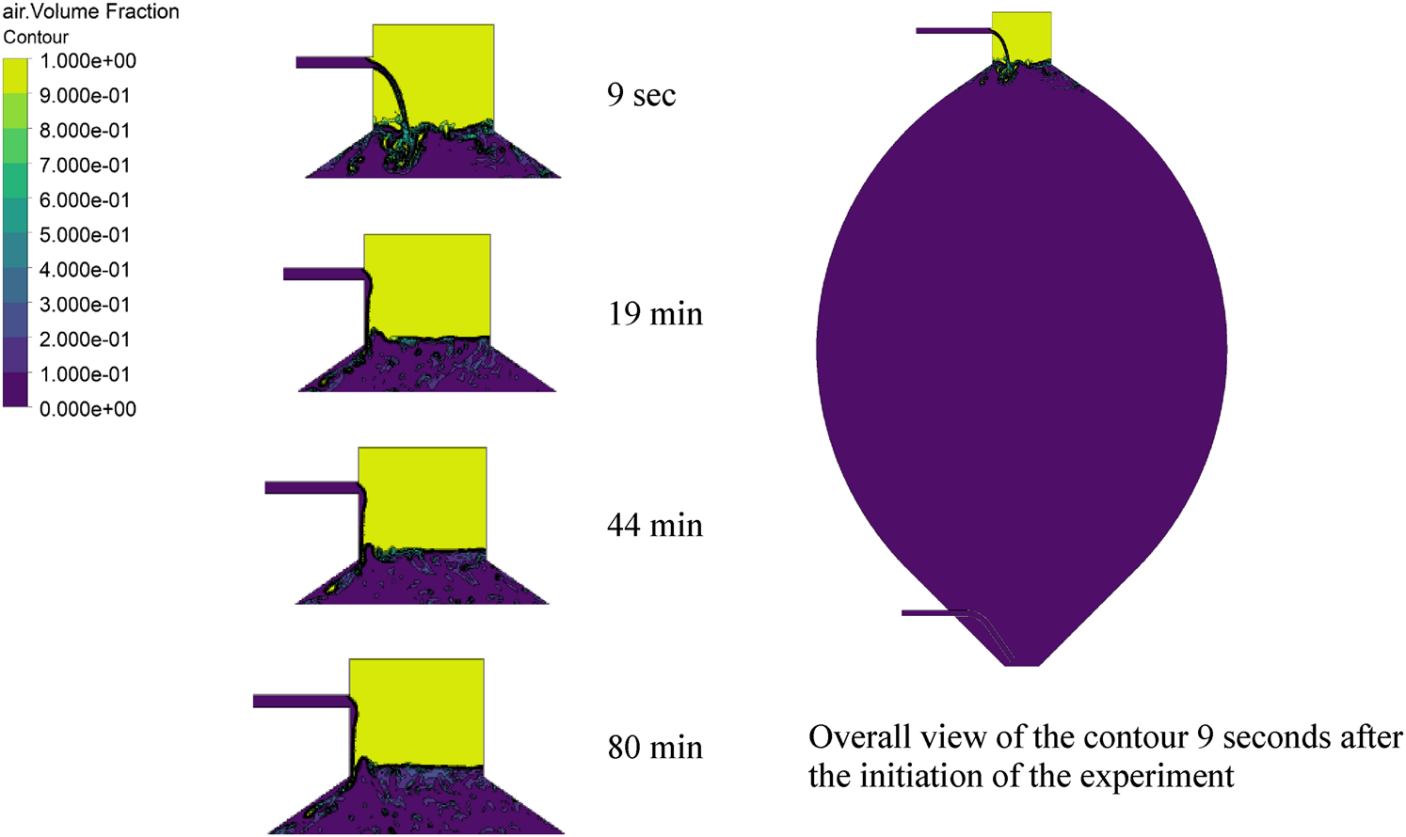
Volume fraction of air above the liquid level and within the slurry flow at different time instants

Figure 6 shows the liquid phase volume fraction contours at instances of 9 s, 19, 44 and 80 min of simulation clock time after the initiation of the simulation. These time steps are selected for a proper comparison between the results of the single- and multi-phase simulations, distributed over 80 min, which is the time required to reach a pseudo-steady state of the flow field.

Figure 6 reveals a deformation in the shape of the sludge which is injected from the top. A splashing inlet BC, next to the relatively low inlet velocity and the increasing pressure at the top of the digester, makes the sludge flow stick to the wall of the tank after approximately 10 s, and then the fluid flow pattern in the upper part remains unaltered.

**Fig. 7:**
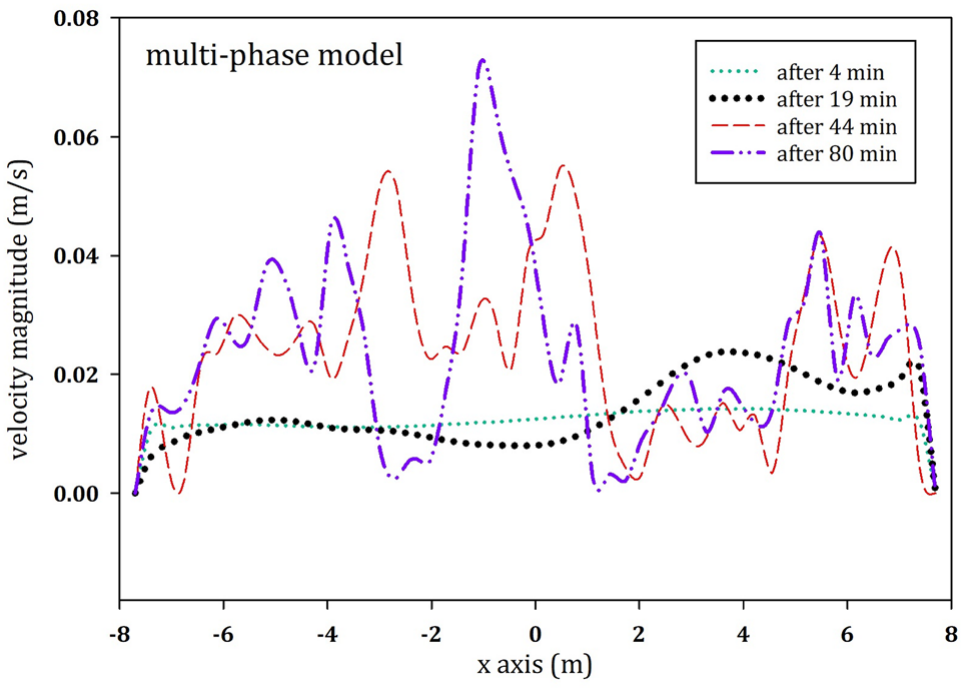
Vertical velocity magnitude along the horizontal axis in the centre of the tank at different time instants for the multi-phase model

Velocity profiles at the cross section in the center of the digester allow for a general assessment of the fluid behavior in the tank. Thus, Fig. 7 depicts the fluctuations of velocity magnitude along the horizontal axis in the center of the tank for the points in time defined above.

Figure 7 shows that the velocity profile at the center of the tank in the multi-phase model does not follow a constant pattern, although a similarity in the shape of the profile develops after 44 min. This can be explained by the weakness of the fluid’s inertial forces at the inlet BC, which leads to temporary flow streams within the tank. However, the range of velocity fluctuation remains below 0.066 m*/s*. After 44 min, we can observe that the regions far from the lateral walls of the digester tend to have a higher velocity magnitude than the regions closer to the walls. A comparison between the profile after 80 min in the multiphase model and the velocity profiles of the single-phase models will be done in Fig. 13.

### Single-phase models

After implementation of the single-phase transient models with three different inlet BC, the results can be presented by visual contours, quantitative plots and other post-processing quantities like mixing time and dead volume zones. In addition, steady-state simulations are conducted.

**Fig. 8:**
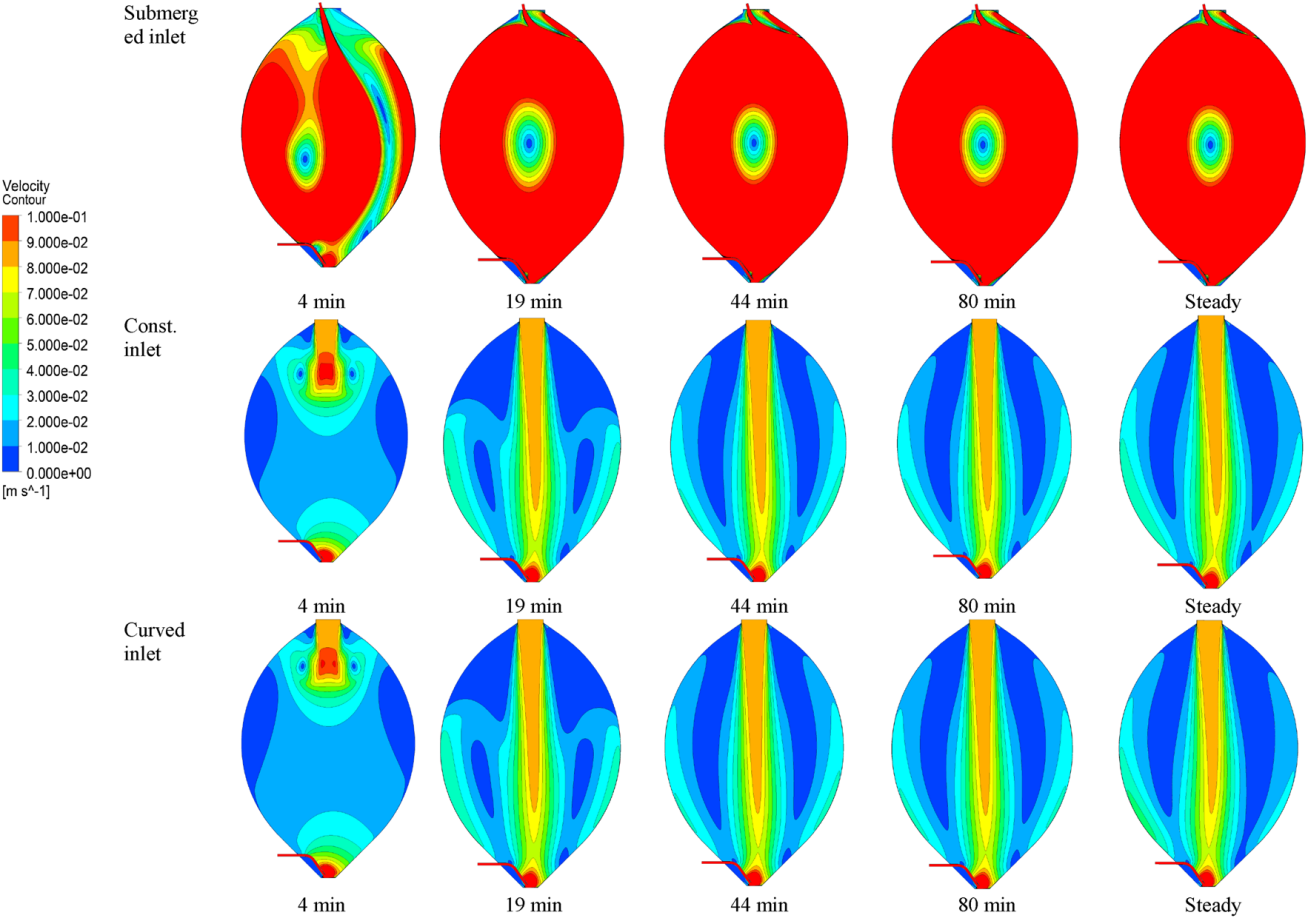
Velocity contours in the cross section of the digester for the submerged inlet, the distributed constant inlet and the distributed curved inlet

Based on the obtained velocity fields in the transient and the steady state simulation, Fig. 8 shows the velocity contours at different time steps for models with submerged inlet, distributed constant inlet and distributed curved inlet configurations.

As shown in Fig. 8, the fluid velocity computed for the model with the submerged inlet BC is much higher than that for the models with distributed constant and curved BCs. This holds true for all time steps and tank regions. Moreover, in all models, the contour patterns after 19 min are in close agreement with the steady state solution. Thus, a simulation time of 80 min proves to be sufficient to reach a steady state.

**Fig. 9:**
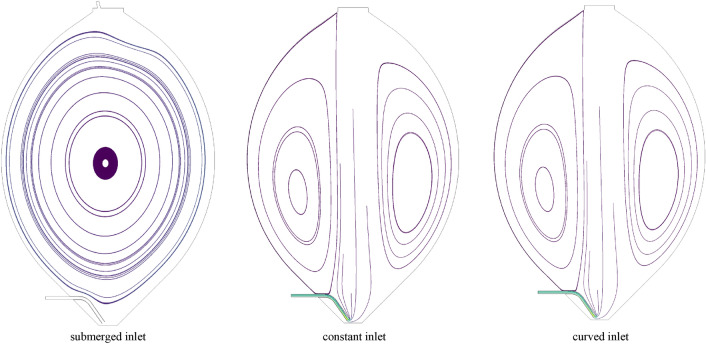
Streamlines in the cross section of the digester for the submerged inlet configuration, the distributed constant inlet configuration and the distributed curved configuration 80 min after the initiation of the simulation

Streamlines, based on the pressure and velocity fields, also help in understanding the fluid behavior within the digester. Thus, streamlines of the three models with various inlet BC, which develop 80 min after the initiation of the simulation, are shown in Fig. 9.

The difference in the case with submerged inlet BC (the higher inlet velocity in submerged inlet, compared to the splashing inlet) results in quite different streamlines patterns as compared to the ones with the distributed constant and the curved inlet conditions (splashing inlet). Additionally, the difference between the velocity contours (Fig. 8) and streamlines (Fig. 9) of the two models with distributed inlet configurations cannot be declared in this scale, therefore more quantitative parameters should be investigated, to distinguish between the model with distributed constant and curved inlet profile.

**Fig. 10:**
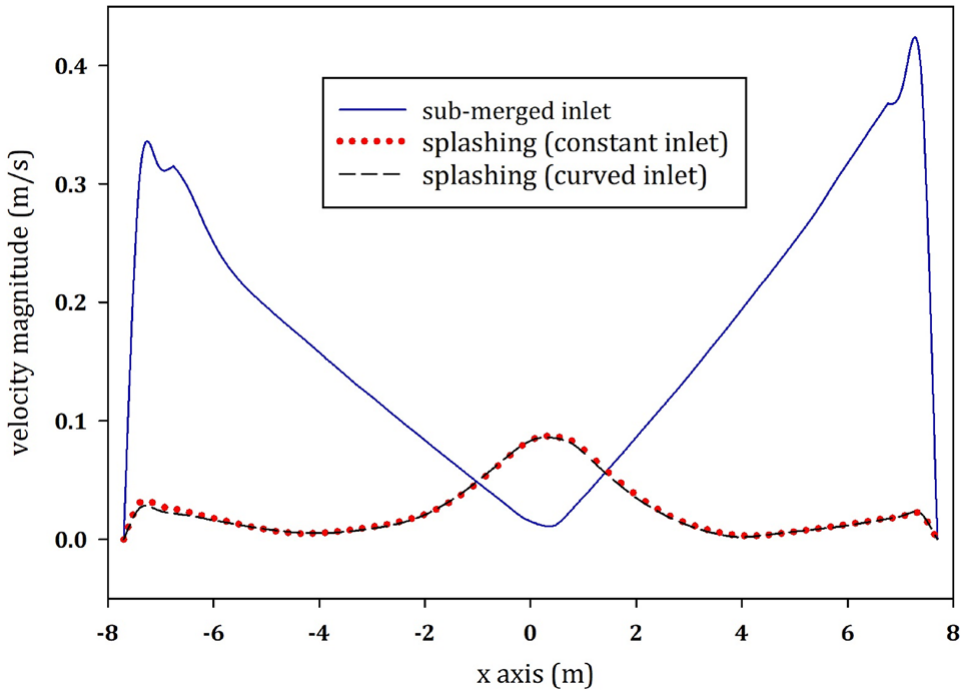
Velocity magnitude along the horizontal axis in the centre of the digester for the submerged inlet, the distributed constant inlet and the distributed curved inlet once a steady state developed

As a quantitative parameter, the velocity magnitude is plotted at the cross section of the tank for each time step. Figure 10 shows the velocity profiles at the center line for the models with submerged inlet, distributed constant inlet and distributed curved inlet, at 4, 19, 44 and 80 min as well as after a steady state is reached.

Figure 10 shows how the shape of velocity fields in Fig. 9 affects the velocity profiles of the models with submerged and splashing inlet configurations at the center. For the case with submerged inlet, both the shape of the plot and range of the plot is different for the models with constant and curved inlets. The maximum velocity magnitude of the steady state solution at the center of the tank is 0.48 m*/s* for the submerged inlet. However, the maximum velocity magnitude for the models with constant and curved inlet is only 0.087 and 0.086 m*/s*, respectively–a mere 18% of the submerged inlet condition. This is because of the difference in the inlet velocity of each model, which has affected the velocity range at the center. Inlet BCs also have an important effect on the shape of the velocity profile in the center. As shown in Fig. 10, due to the high submerged inlet velocity, the shape of velocity profile in the model with submerged inlet is different than for the constant and curved inlets. The velocity is higher in the model at the middle of the plot for the models with constant and curved inlets, whereas the velocity is higher near the walls for the model with submerged inlet. The reason of this behavior becomes clear from Figs. 8 and  9, as in the case with the submerged inlet a considerable circular flow is formed within the tank. However, for each case, with either constant or curved inlet BC, there is a dominant downward flow in the center of the tank and a weaker upward flow near the walls.

### Mixing time analysis

**Fig. 11:**
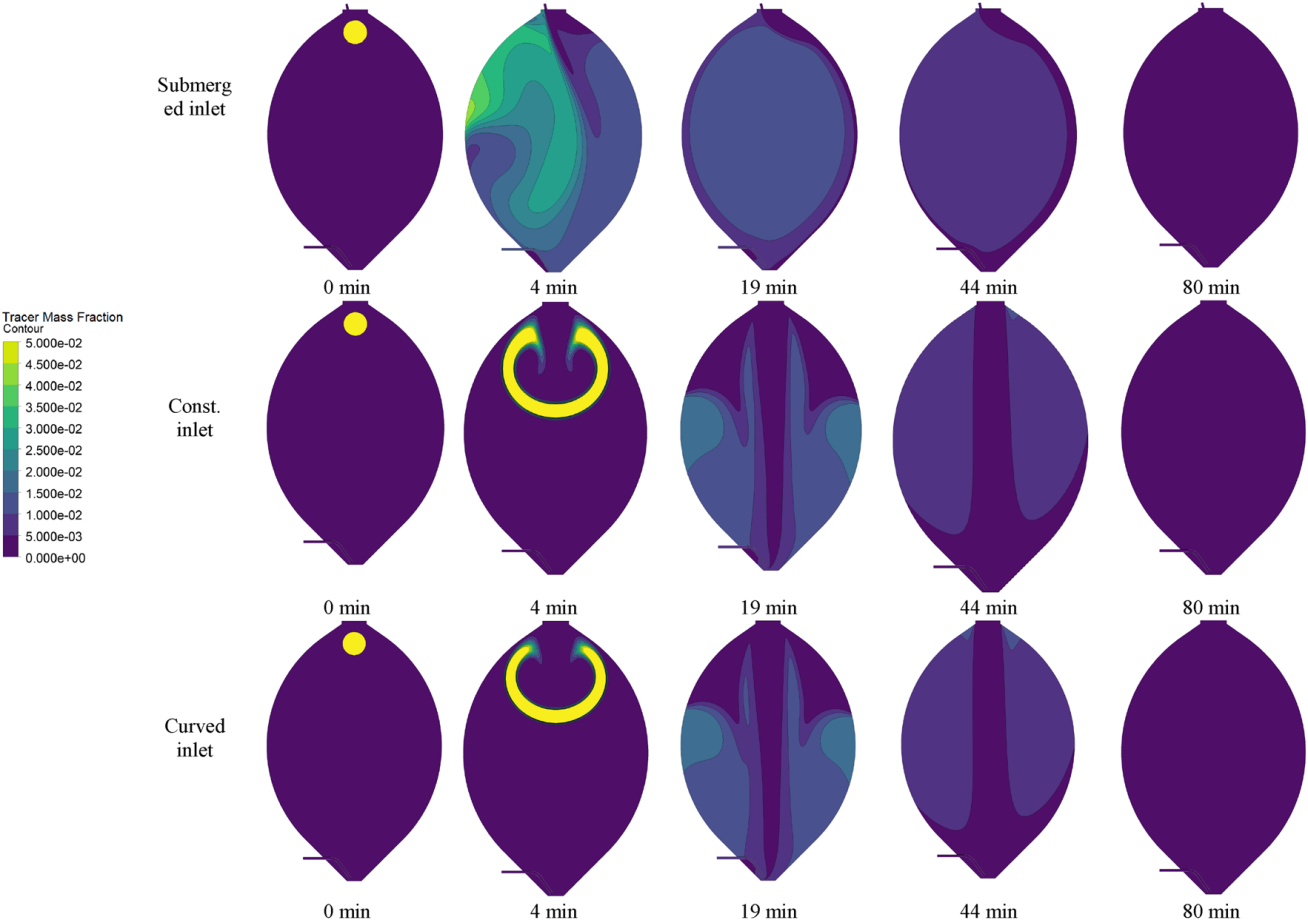
Contours of the tracer mass fraction in the cross section of the digester for the submerged inlet, the distributed constant inlet and the distributed curved inlet

The definition of mixing time is an important factor in the analysis of recirculation. For the calculation of the mixing time, we follow the procedure according to [21, 22]. We define a circular region at a specific location in the digester tank, which has a radius of 1 m and is located 9 m above the center of the tank (cf. Fig. 11). This zone is filled with a tracer fluid with equal density and viscosity as the main fluid. This tracer fluid is tracked while the simulation runs. Figure 11 shows the distribution of the tracer compound within the digester for the three different models. It depicts the tracer mass fraction contours at a resolution of 0.005.

Figure 11 depicts that the concentration of the tracer mass diminishes after only 4 min for the submerged inlet configuration. At this time instant the tracer is already fully distributed within the tank. Figure 11 also reveals that the model with the submerged inlet predicts quite different flow patterns than the models with constant and curved inlets. One possible explanation is the high inlet velocity originating from the focused submerged flow stream. In comparison, the distribution of the tracer within the tank is significantly slower in the models with distributed constant and curved inlet configurations. 80 min after the initiation of the simulation the tracer mass fraction drops to below 0.005 for all models.

**Fig. 12:**
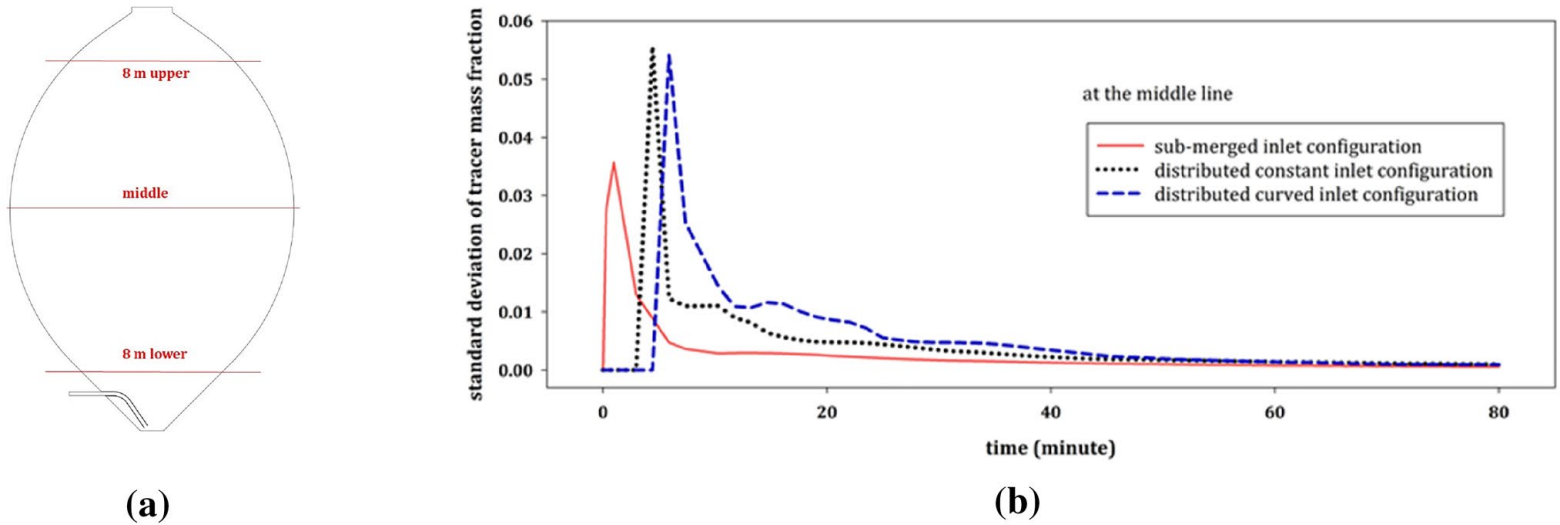
The three analyzed cross sections, located 8 m above, at and 8 m below the centre of the tank (**a**) and the evaluation of the standard deviation of the tracer mass fraction at the centre line to analyze the mixing time 80 min after the initiation of the simulation for the submerged inlet, the distributed constant inlet and the distributed curved inlet (**b**)

In addition, the standard deviation of the tracer fluid concentration is observed at three cross sections to assess the state of mixing in different regions of the tank. As shown in Fig. 12(a), the first cross section is located 8 m above the center of the tank, the second is located at the center itself and the third cross section is placed 8 m below the center of the tank. This makes it possible to estimate the time for the initial step of mixing from top to the bottom of the digester. Here we assume that mixing reaches a steady state once the standard deviation of the tracer concentration drops below 0.001. Figure 12(b) illustrates the standard deviation which is evaluated at the center line and for each model.

Figure 12 shows that the mixing time in the model with submerged inlet is much lower than for the other two models. Similar results are observed by analyzing the standard deviation of the tracer fluid concentration at the two other lines, 8 m above and 8 m below the center. This is because in the submerged inlet configuration, the injection of the fluid into the domain with a high inlet velocity results in an imperative agitation within the tank.

#### Energy consumption analysis

In order to analyze the energy consumption which is required for the agitation of the tank for each inlet configuration, we use the method by Bridgeman [9]. The energy dissipation rate is calculated by using the turbulence closure model. Therefore, the overall power consumption is estimated by numerical integration of the local power consumption over the entire volume of the tank, as in Eq. 10:

10 $$P=\rho \int \varepsilon dV$$

where $\rho$ is the density, $\varepsilon$ denotes the energy dissipation rate and $V$ indicates the volume. According to the results, the power consumption for the model with the submerged inlet is 208% and 212% higher than the models with distributed constant and curved inlet configuration, respectively.

## Discussion

All simulations are run on a computer with 16 GB RAM, and a Core (TM) i7 CPU of 3.2 GHz, five cores of which are used in parallel. The simulation performance, which includes the simulation clock time and the time step used, is summarized in Table 3. The time steps are dynamically controlled by a Courant number criterion.

**Table 3:** running properties during the simulation

	Splashing inlet configuration			Submerged inlet
	Two-phase	Single-phase (distributed constant inlet)	Single-phase (distributed curved inlet)	
Time step size	0.01–0.1 s	0.01–0.1 s	0.01–0.1 s	0.01–0.1 s
Iterations per time step	50	30	30	30
Simulation clock time	12 days	2 days	2 days	2 days

For all models the standard deviation of the tracer mass fraction in the upper cross section in the tank fluctuates with a higher domain, which is due to the proximity to the tracer region. While the values do not differ significantly, the standard deviation for submerged inlet is lower, which indicates that the submerged inlet results in a better agitation in the upper region.

Likewise, the numerical results suggest that the tracer reaches the center cross section much faster in case of the submerged inlet (after 3 min) as in the case of the splashing inlet (9 min for the constant inlet and 8 min for the distributed inlet). This reveals again that the agitation in the digester is higher in the model with the submerged inlet as compared to the other cases.

A sufficient level of mixing is obtained with all models after 52 min. However, in order to obtain a homogeneous tracer mass concentration, simulations are continued until 80 min after initiation. At this time the concentration of the tracer mass fraction is below 0.005 at each point within the tank. The mixing time is not the only criterion to assess the digester mixing. Another significant criterion is the contribution of the dead volume [15] which should be investigated to gain an insight into the mixing performance.

The dead volume threshold is computed by the method in [15], where they defined dead zones as regions with velocity magnitudes less than 0.02 m*/s*. We employ a user-defined memory, based on the 'DEFINE_ADJUST' function in ANSYS Fluent, to compute the total volume of cells with a velocity magnitude less than 0.02 m*/s*, which are considered as stagnant zones (details are given in the Supplementary material file—Sect. 2).

The results show that in the steady state, the amount of dead volume is 0.3% for the submerged inlet model. However, for the models with distributed constant inlet and curved inlet, the amount of dead volume is 28% and 27%, respectively. This result confirms the findings above that the submerged inlet better mixes the digester.

**Fig. 13:**
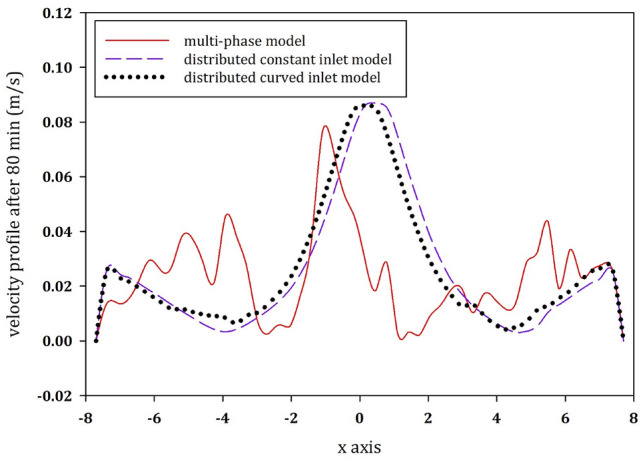
Comparison of the vertical velocity magnitudes along the horizontal axis in the center of the tank for the splashing models (single-phase models with distributed constant inlet and the distributed curved inlet) and the multi-phase model 80 min after the initiation of the simulation

It is well understood that the real physical phenomena would require a transient multi-phase CFD simulation. However, due the model complexity and the excessive CPU requirement, mixing in anaerobic digesters is frequently modeled as a single-phase model. In order to distinguish between the best matching single-phase model and the multi-phase model, we compare the results of the single-phase models with the multi-phase ones. These results are depicted for the velocity profile at the center cross section of the tank 80 min after initiation of the simulation (see Fig. 13).

For the models emulating splashing inlet configurations, Fig. 13 depicts that the models with distributed constant and curved inlet conditions are in good agreement with the results of the multi-phase model. There is an 81% and a 75% difference between the multi-phase model and the models with distributed constant and curved inlet, respectively. In conclusion, the single-phase model with the distributed curved inlet condition is a good approximation of a multi-phase simulation with the splashing inlet configuration.

Based on Fig. 10, it can be observed that the behavior of fluid flow within the digester changes by switching the type of inlet velocity from splashing to directly injecting sludge recirculation. Although splashing inlet configuration brings about a high amount of dead volume (27% of the digester tank), submerged inlet configuration (with directly injecting sludge) assures proper mixing of the fluid flow inside the tank. Submerged inlet has a higher amount of inertial force, which leads to sludge recirculation with a maximum velocity of above 0.4 m*/s*. The submerged inlet configuration results in the reduction of the dead volume by 98%.

Understanding the capability of sludge recirculation in mixing can obviate the need for any other external mixing method, e.g. draft tube mixer or gas injection. This study shows that, although the power consumption for directly injecting the sludge within the tank in the submerged inlet configuration is around three times as much as compared to splashing inlet configuration, it eliminates the need for any other types of external mixer. This enhances the overall energy proficiency of the digester. The amount of biogas yield depends upon multiple factors, including the quality of mixing [39], which can also be analyzed through the energy dissipation rate [40] and subsequently energy consumption. According to [40] as long as the energy dissipation rate lies below 170 W*/m* (like in our case), biogas yield stands within the optimum range.

## Conclusions

This study investigates the effect of two different inlet configurations on anaerobic digester mixing, that is the splashing inlet configuration (simulated with a detailed two-phase model and subsequently with two single-phase models) and the submerged inlet configuration (simulated with a single-phase model). For validation, the resulting velocity profiles are compared to a previous study which investigated the same digester. Following results are concluded:

- All simulation results agree that the fluid dynamics in the digester are highly dependent on the inlet configuration.
- From the point of mixing efficiency and in order to keep the dead volume minimal, the fluid should be injected directly into the enclosed fluid domain of the tank.
- The submerged inlet, which is conveniently modelled by means of a single phase model, reveals a substantial mixing effect and–for the case study–a negligible amount of dead volume in the tank. This is due to the high inlet velocity, which leads to higher inertial forces within the tank, as compared to the situation with a splashing inlet configuration.
- A splashing inlet configuration can be computed in detail with two-phase models but also emulated in single-phase conceptually applying a distributed inlet. However, by employing single-phase models with distributed inlet configuration for emulating the two-phase physics of the splashing inlet, the simulation clock time is reduced to 15%.
- For emulating recirculation mixing with a splashing inlet, the distributed curved inlet BC offers the closest results to the multi-phase simulation but for practical purposes the constant distributed inlet is sufficient.
- The power consumption for injecting the sludge within the tank in the submerged inlet configuration is around three times as much as compared to splashing inlet configuration.

## Supplementary Information

Below is the link to the electronic supplementary material.

Supplementary file1 (DOCX 3756 kb)
