# Supplementary material for: On the effect of the inlet configuration for anaerobic digester mixing
Source: Bioprocess Biosyst Eng. 2021 Jul 21;44(12):2455–68. doi: 10.1007/s00449-021-02617-4 (PMC8536570; doi:10.1007/s00449-021-02617-4)
Supplement: Supplementary file 1 — Supplementary file1 (DOCX 3756 kb) [file 449_2021_2617_MOESM1_ESM.docx]

**Supplementary material file for the paper *on the effect of the inlet configuration for anaerobic digester mixing***

Soroush Dabiri^a,^*, Johannes Sappl^a^, Prashant Kumar^a^, Michael Meister^b^, Wolfgang Rauch^a^

^a^Unit of Environmental Engineering, University of Innsbruck, Innsbruck, Austria

^b^Department of Environmental, Process, and Energy Engineering, Management Center Innsbruck, Innsbruck, Austria

Corresponding author: [Soroush.dabiri@uibk.ac.at](mailto:Soroush.dabiri@uibk.ac.at), Tel: +43 512 507 62104

1. **Geometry and mesh**

Weir pipes location:

Weir pipes positioned at the upper part of digester tank. Figure 1 show the location of weir pipes. In the 3D reality of the digester, they do not have a significant effect on the hydrodynamics, while within the 2D platform they will play a more important role. Thus the effect of weir pipes are neglected within the geometry designed for modelling.


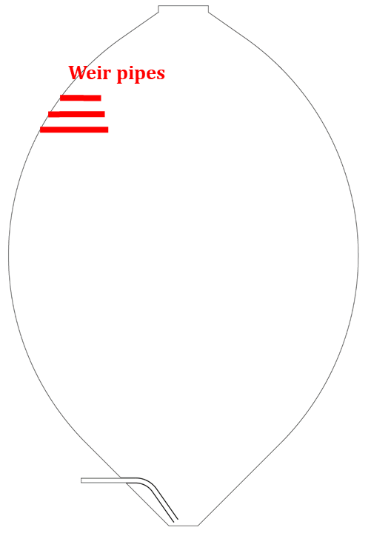


Figure 1 the location of weir pipes within the schematic shape of the digester

A full geometry of two phase with the phases

Figure 2 shows the geometry, designed for the two-phase simulation for simulating the splashing inlet configuration, including the sludge liquid phase (indigo) and biogas phase (yellow).


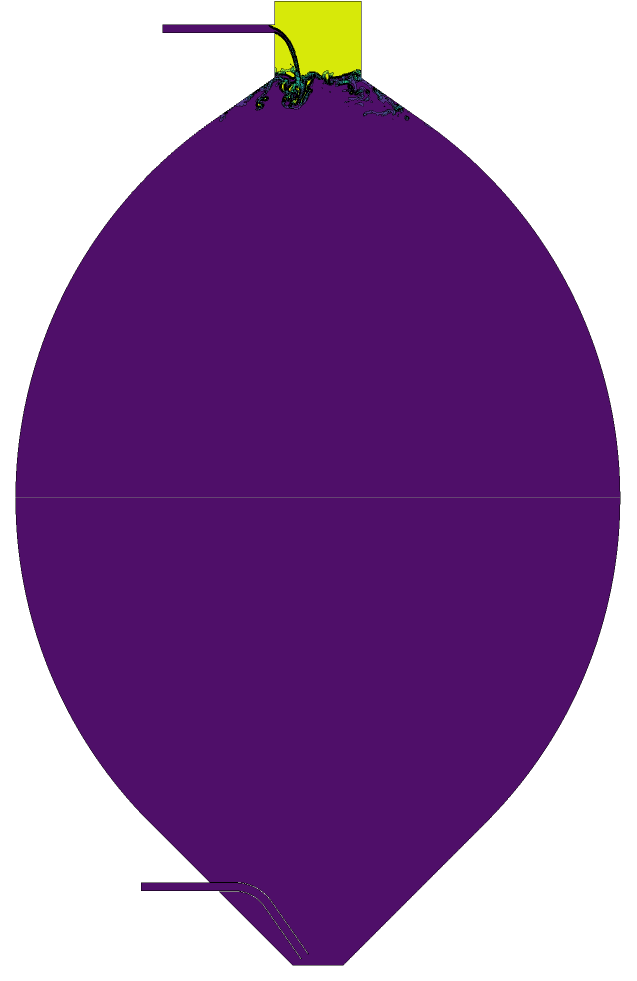


Figure 2 the geometry of the two-phase splashing inlet configuration

Mesh:

As a sample, the final mesh network of the model for simulating splashing inlet configuration in two-phase is depicted by Figure 3.


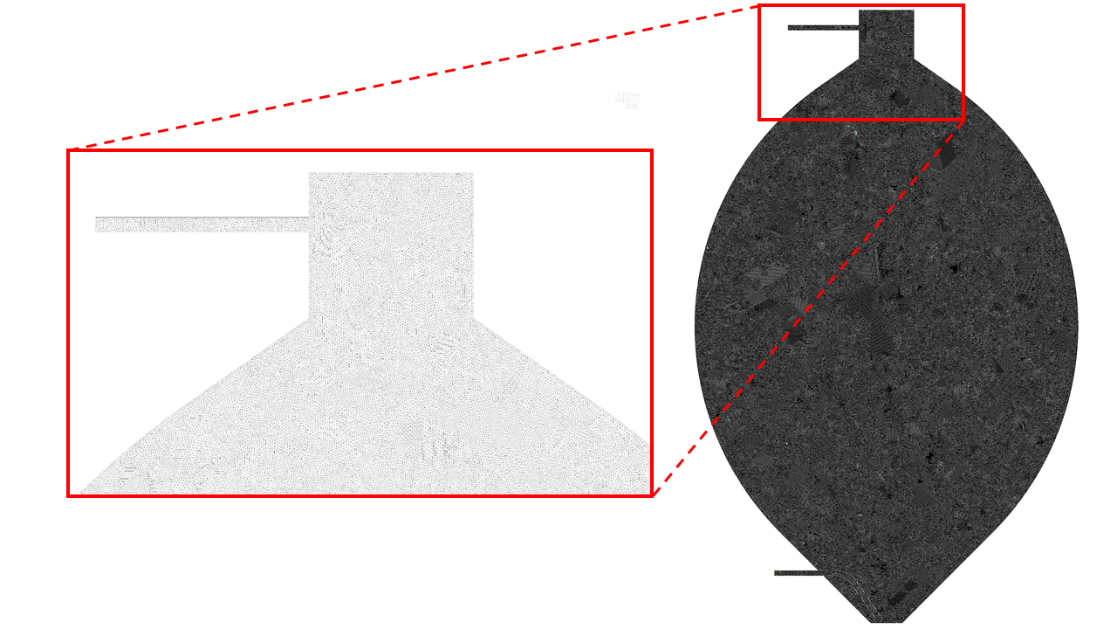


Figure 3 the final mesh network of the model for simulating splashing inlet configuration

1. **Methods**

Setting the distributed curved inlet configuration:

ANSYS Fluent has the option to impose non-constant velocity profile. This is possible through user defined functions. For doing so, the plot of velocity profile at the location 30 cm below the sludge surface is extracted from the multiphase simulation, and then the function of velocity based on the horizontal location is extracted. The udf code for inlet non-constant inlet profile is in the following.

| /***********************************************************************  vprofile.c  UDF for specifying steady-state velocity profile boundary condition  ************************************************************************/  #include "udf.h"  DEFINE_PROFILE(inlet_y_velocity, thread, position)  {  real y[ND_ND]; /* this will hold the position vector */  real a;  face_t f;  a = y[0];  begin_f_loop(f,thread)  {  F_CENTROID(y, f, thread);  F_PROFILE(f, thread, position) = 0.75*pow(1500,-1)*(-0.93*pow(a,8)+7.4*pow(a,7)-23*pow(a,6)+37*pow(a,5)-30*pow(a,4)+13*pow(a,3)-2.3*pow(a,2)+0.17*a+0.001);  }  end_f_loop(f, thread) |
| --- |

This code is interpreted by the software itself and does not need any external compiling tool.

Calculating dead volume:

In order to calculate the volume, in which the velocity magnitude within the cells are below 0.02 m/s, we need to specify a memory to the accummulative volume of the cells with a velocity magnitude of below this criteria.

For doing so the following code is written:

| #include "udf.h"  #define vel_min 0.02  DEFINE_ADJUST(my_deadvolume, domain)  {  real deadvol=0;  Thread *cell_thread;  cell_t cell;  thread_loop_c(cell_thread, domain)  {  begin_c_loop(cell, cell_thread)  {  real u, v, vel;  u=C_U(cell, cell_thread);  v=C_V(cell, cell_thread);  vel=sqrt(pow(u,2)+pow(v,2));  if(vel<vel_min)  {  deadvol += C_VOLUME(cell, cell_thread);  }  }  end_c_loop(cell, cell_thread)  }  printf("the value of dead volume is %f\n", deadvol);  } |
| --- |

Afterwards this code is interpreted by the software, and imposed into the simulation. Therefore, the solver calculates the amount of dead volume after each iteration.

1. **Mesh sensitivity analysis**

The parameters for calculating mesh sensitivity for the case with distributed inlet configuration are summarized in Table 1.

Table 1 the calculation of the parameters for assessing mesh sinsitivity

| Parameter | Unit | Definition | Value |
| --- | --- | --- | --- |
| A | m^2^ | Area of the tank | 251.85 |
| h_1_ | m | Grid size | 0.050 |
| h_2_ |  |  | 0.025 |
| h_3_ |  |  | 0.015 |
| Φ_1_ | m/s | Velocity magnitude at a specific central point | 8.56*10^-2^ |
| Φ_2_ |  |  | 8.30*10^-2^ |
| Φ_3_ |  |  | 8.24*10^-2^ |
| ε_21_ | m/s | Difference in the velocity magnitude at a specific central point | 0.26*10^-2^ |
| ε_32_ |  |  | 0.06*10^-2^ |
| r_21_ |  | Ratio of two grid sizes in sequence | 2.00 |
| r_32_ |  |  | 1.66 |
| p |  | Order of the convergence | 1.44 |
| $e_{a}^{21}$ |  | Relative error | 3.03*10^-2^ |
| ${GCI}_{fine}^{21}$ |  | Grid convergence index | 2.21*10^-2^ |
| $e_{a}^{32}$ |  | Relative error | 0.72*10^-2^ |
| ${GCI}_{fine}^{32}$ |  | Grid convergence index | 0.83*10^-2^ |

Besides calculating GCI, it is necessary to conduct a grid independence study over the velocity magnitude profile. After obtaining the velocity profiles for the three meshes, the difference in velocity profiles is captured by the velocity magnitude at 50 points which are evenly distributed within the centred line, and calculating the mean of velocity difference, σ, for all the points as follows:

| $\sigma_{i}=100\frac{(\acute{V}_{i}-V_{i})}{V_{i}}$ | (1) |
| --- | --- |

where $V_{i}$is the velocity magnitude of the point i in for one grid network, compared to $\acute{V}_{i}$of the similar point in the other grid. For distributed inlet conditions, grid sizes with element numbers from 100,644 and 404,878 to 1,117,855 elements are investigated.


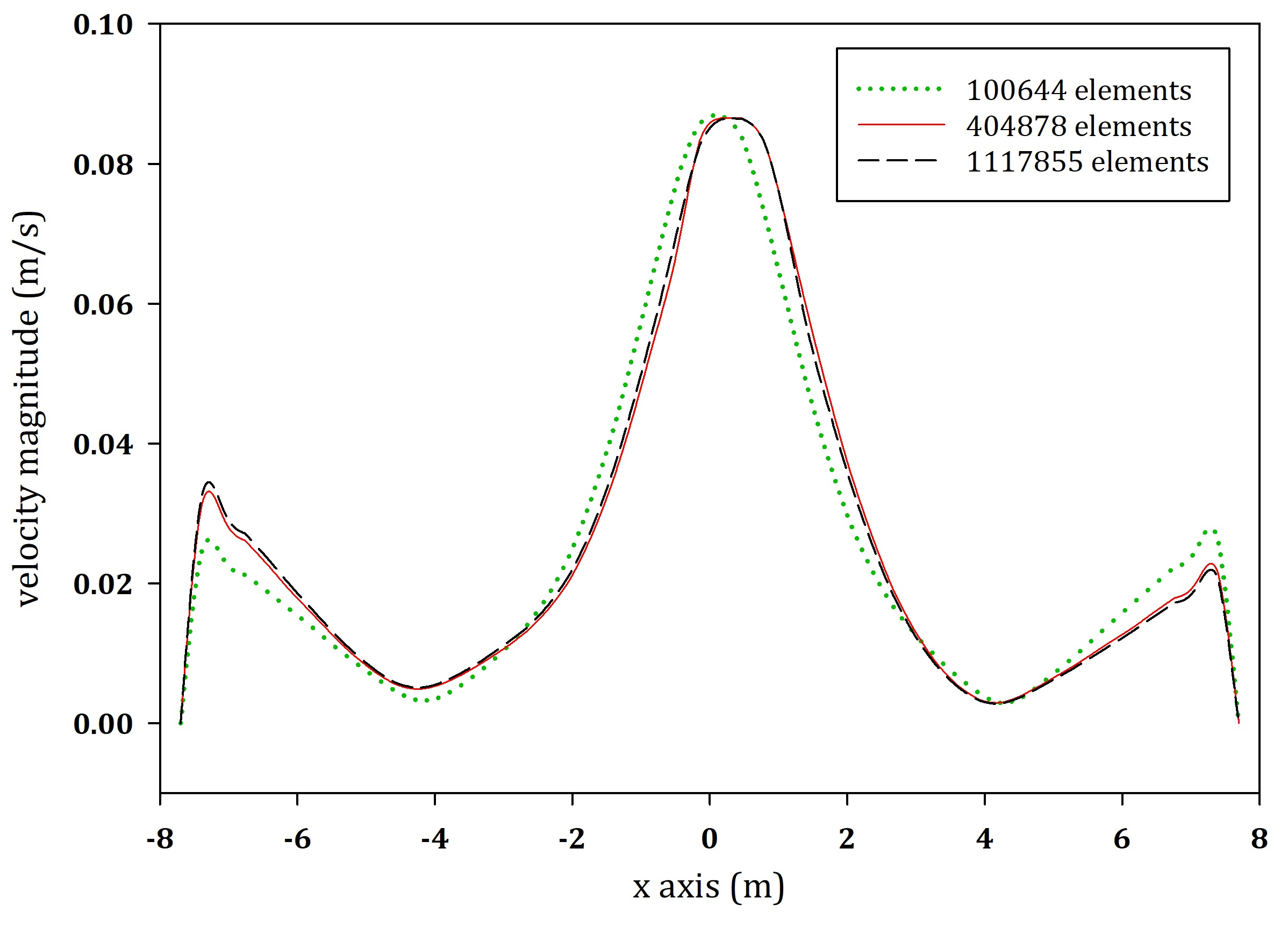


Figure 4 Mesh independency study of the vertical velocity magnitude as a function of the horizontal axis located at the centre of the tank, for the distributed inlet configuration

As Figure 4 depicts, in the model with the distributed inlet configuration, the mesh with the lowest number of elements (i.e. 100,664) needs to be discarded since it substantially (15.7%) deviates from the higher resolution with 404,878 elements. On the other hand, a further increase to 1,117,855 improves the accuracy only by 3.6%. Hence, the mesh with 404,878 elements is chosen for the analysis. A similar procedure is implemented for the model with the submerged inlet.

1. **The difference between 2D and 3D**

The 2D and 3D geometries designed for the comparison with each other are depicted by Figure 5.

| 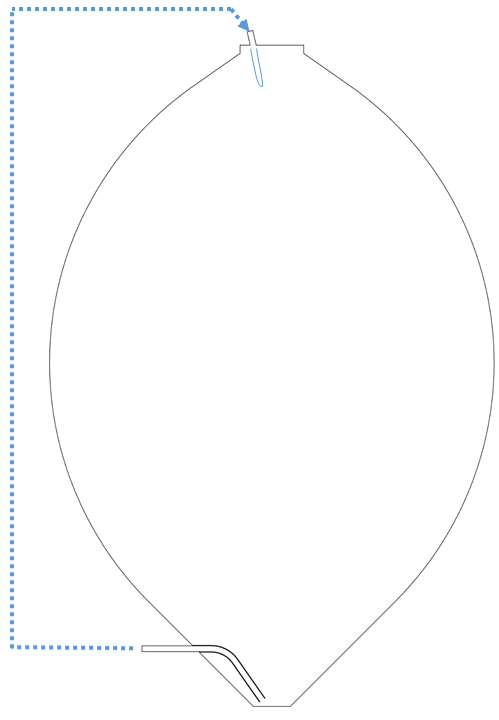 | 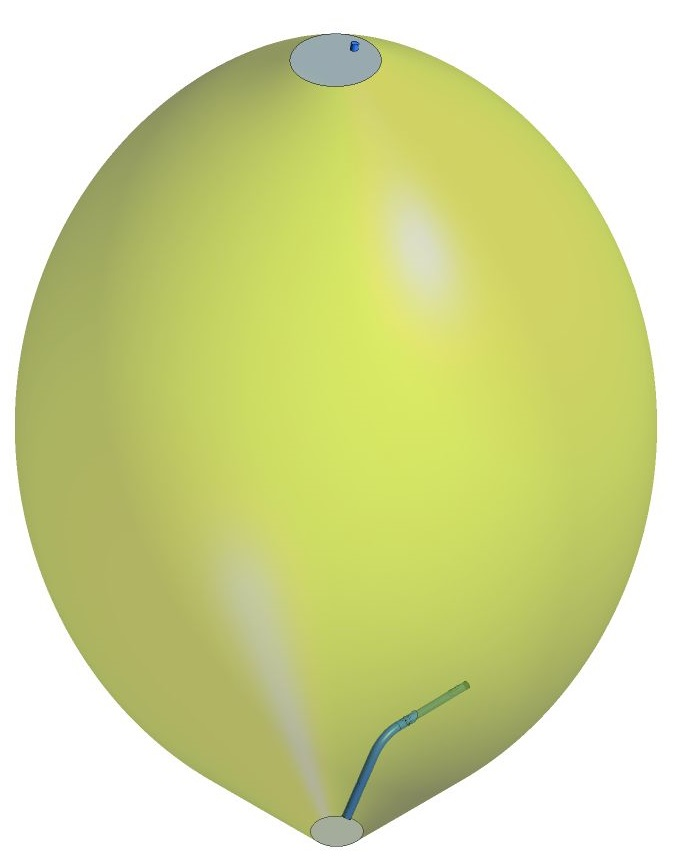 |
| --- | --- |

Figure 5 The 2D and 3D geometries of the digester, designed to compare the results of the 2D and the 3D models

After analysing the optimum amount of mesh elements for each model, the fluid flow equations were solved. They are continiuty, Navier-Stokes and turbulence-related equations, which weere solved for each model in steady state. the residuals convereged to the order of 10^-5^ for the models. Figure 6 shows the velocity contours of the results. Authors warn that the match of the results between the 2D and the 3D platform is not happenning in all parts of the case. This is because of the difference between the 2D and the 3D platforms.

| 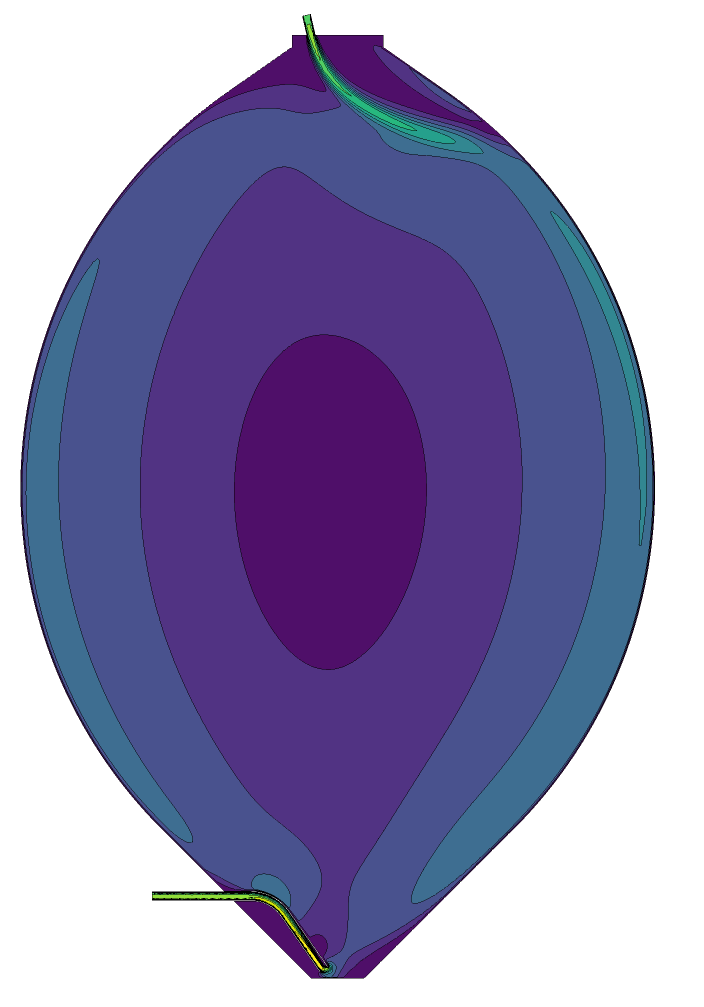 | 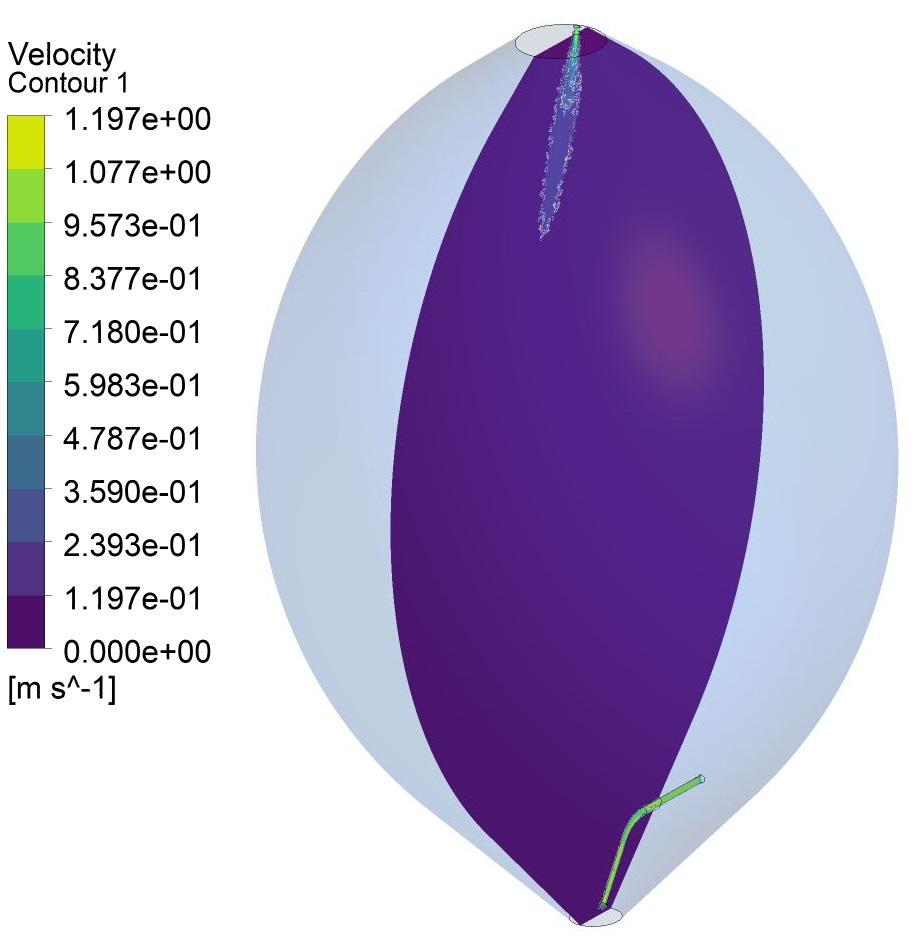 |
| --- | --- |

Figure 6 the Velocity contours of the 2D and the 3D model models

1. **References (Previous models)**

Figure 7 shows the geometry and the results of the model that Meister et al. in [1] have used. They have used a geometry containing a draft tube, due to the previous facilities in Achental-Inntal-Zillertal (AIZ) wastewater treatment plant at the time.

| 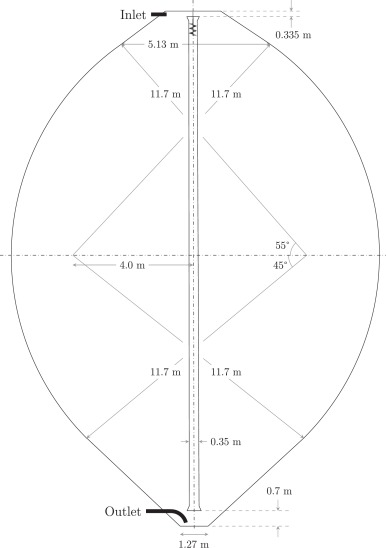 | 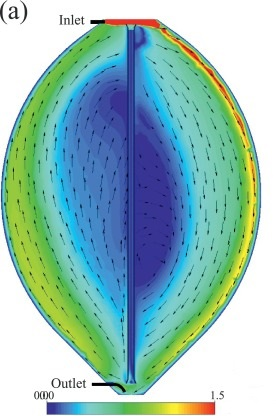 | 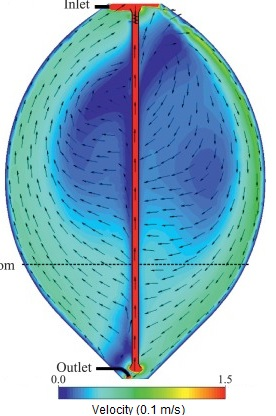 |
| --- | --- | --- |
| (a) | (b) | (c) |

Figure 7 The geometry of the AIZ digester (a), Velocity contours of the AIZ digester operated solely with pumped recirculation are shown for the plane parallel when the TS concentration is 2.5% (b), velocity contour of the AIZ digester with draft tube and the pumped recirculation (c)

After conducting the CFD simulation, their findings are as follows:

- The mode of operation with pumped recirculation and impeller induced mixing yields the highest level of mixing.
- The axial symmetry of the flow pattern is suspended by the pumped recirculation, but the overall velocity magnitudes increase.
- The pumped recirculation in absence of any impeller induced agitation efficiently mixes the plane parallel to the feeding pipe, while the plane perpendicular to it remains poorly mixed.
- By carrying out the simulations for various total solids (TS) concentrations, they suggested that an increased impeller agitation speed of N=1000 rpm is recommended for a TS concentration of 12.1% to maintain a high level of mixing.

Figure 8 depicts the geometry results of Rezavand et al. in [2], investigating the same wastewater treatment plant. However, they mostly focused on modeling the biokinetics of the digester in a particle-based platform, the gpuSPHASE software.


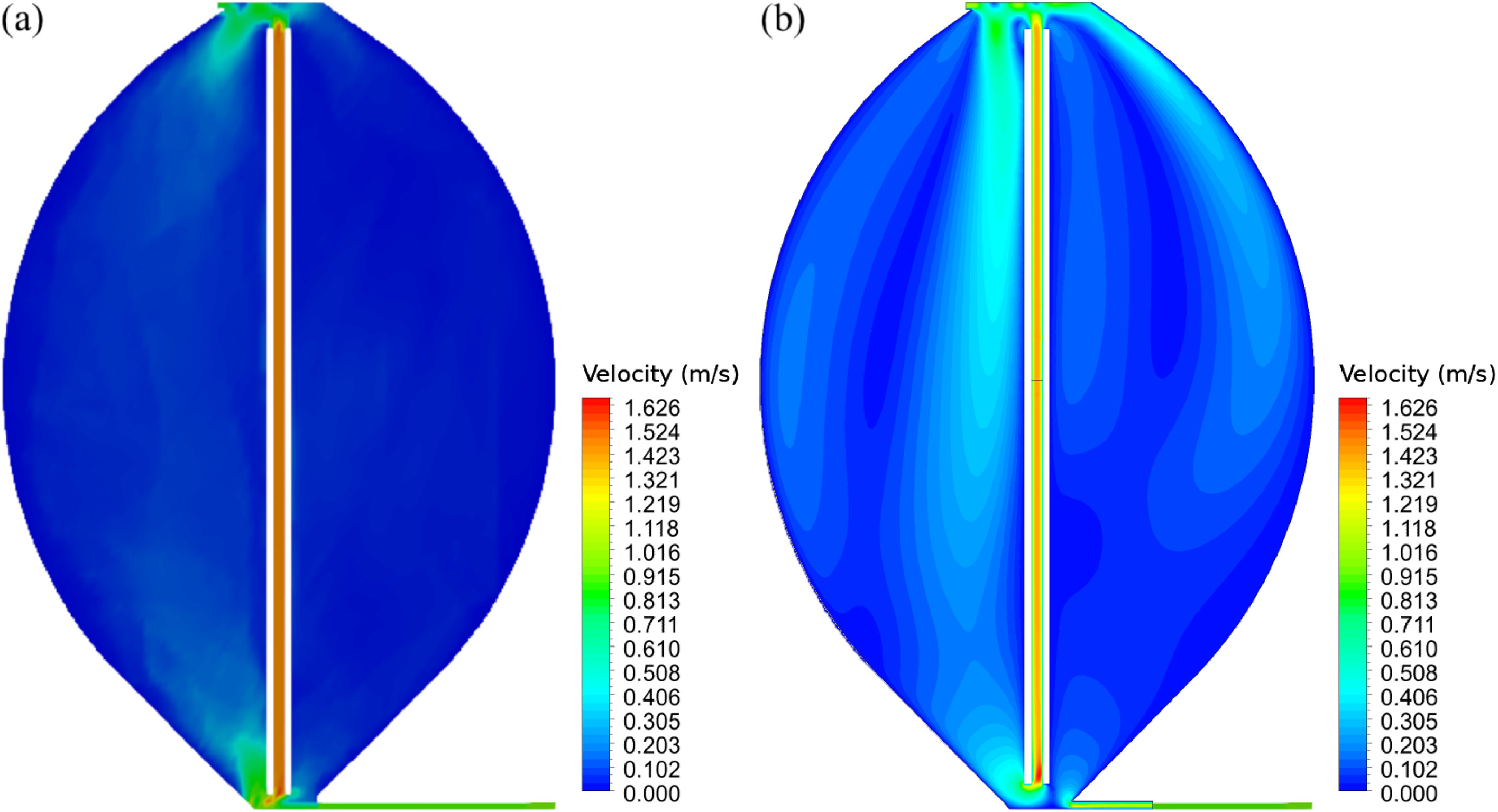


Figure 8 Contours of the velocity field predicted by (a) SPH and (b) 2D ANSYS Fluent for the real life egg-shaped anaerobic digester.

Their results are summarized as follows:

- With the use of smooth particle hydrodynamics (SPH) method, it can be possible to directly analyze the mixing effect on biogas production.
- The biological concentrations can be spatially resolved due to the particle-based disrcretization of the computational domain.
- The peculiarities of SPH in dealing with multi-phase problems facilitate the description of the biokinetics.
- Through application of the model to a real world egg-shaped anaerobic digester, it has been shown that the model could successfully replicate both mixing and biochemical processes. However, industrial application of the proposed model depends on computational efficiency of the future SPH solvers.

[1] Meister M, Rezavand M, Ebner C, Pümpel T, Rauch W (2018) Mixing non-Newtonian flows in anaerobic digesters by impellers and pumped recirculation. Advances in Engineering Software 115, 194-203.

[2] Rezavand M, Winkler D, Sappl J, Seiler L, Meister M, Rauch W (2019) A fully Lagrangian computational model for the integration of mixing and biochemical reactions in anaerobic digestion. Computers & Fluids 181, 224-235.
